# Iron deficiency during pregnancy is associated with a reduced risk of adverse birth outcomes in a malaria-endemic area in a longitudinal cohort study

**DOI:** 10.1186/s12916-018-1146-z

**Published:** 2018-09-20

**Authors:** Freya J. I. Fowkes, Kerryn A. Moore, D. Herbert Opi, Julie A. Simpson, Freya Langham, Danielle I. Stanisic, Alice Ura, Christopher L. King, Peter M. Siba, Ivo Mueller, Stephen J. Rogerson, James G. Beeson

**Affiliations:** 1 Burnet Institute, Maternal and Child Health Program, Life Sciences and Public Health, Melbourne, VIC 3004 Australia; 2 Department of Epidemiology and Preventive Medicine, Monash University, Melbourne, VIC 3008 Australia; 3 Centre for Epidemiology and Biostatistics, Melbourne School of Population and Global Health, The University of Melbourne, Melbourne, VIC 3010 Australia; 4 Department of Infectious Diseases, Central Clinical School, Monash University, Melbourne, VIC 3004 Australia; 5 Department of Immunology, Monash University, Central Clinical School, Melbourne, VIC 3004 Australia; 6 Institute for Glycomics, Griffith University, South Brisbane, QLD 4101 Australia; 7 Papua New Guinea Institute of Medical Research, Goroka, EHP Papua New Guinea; 8 Center for Global Health and Diseases, Case Western Reserve University, Cleveland, OH USA; 9 Walter and Eliza Hall Institute of Medical Research, Parkville, VIC 3050 Australia; 10 Department of Parasites and Insect Vectors, Institut Pasteur, 75015 Paris, France; 11 Department of Medicine (RMH), The University of Melbourne, Parkville, VIC 3010 Australia

**Keywords:** Iron deficiency, Low birth weight, Preterm birth, Malaria, Anaemia, Pregnancy, *Plasmodium falciparum*

## Abstract

**Background:**

Low birth weight (LBW) and preterm birth (PTB) are major contributors to infant mortality and chronic childhood morbidity. Understanding factors that contribute to or protect against these adverse birth outcomes is an important global health priority. Anaemia and iron deficiency are common in malaria-endemic regions, but there are concerns regarding the value of iron supplementation among pregnant women in malaria-endemic areas due to reports that iron supplementation may increase the risk of malaria. There is a lack of evidence on the impact of iron deficiency on pregnancy outcomes in malaria-endemic regions.

**Methods:**

We determined iron deficiency in a cohort of 279 pregnant women in a malaria-endemic area of Papua New Guinea. Associations with birth weight, LBW and PTB were estimated using linear and logistic regression. A causal model using sequential mediation analyses was constructed to assess the association between iron deficiency and LBW, either independently or mediated through malaria and/or anaemia.

**Results:**

Iron deficiency in pregnant women was common (71% at enrolment) and associated with higher mean birth weights (230 g; 95% confidence interval, CI 118, 514; *p* < 0.001), and reduced odds of LBW (adjusted odds ratio, aOR = 0.32; 95% CI 0.16, 0.64; *p* = 0.001) and PTB (aOR = 0.57; 95% CI 0.30, 1.09; *p* = 0.089). Magnitudes of effect were greatest in primigravidae (birth weight 351 g; 95% CI 188, 514; *p* < 0.001; LBW aOR 0.26; 95% CI 0.10, 0.66; *p* = 0.005; PTB aOR = 0.39, 95% CI 0.16, 0.97; *p* = 0.042). Sequential mediation analyses indicated that the protective association of iron deficiency on LBW was mainly mediated through mechanisms independent of malaria or anaemia.

**Conclusions:**

Iron deficiency was associated with substantially reduced odds of LBW predominantly through malaria-independent protective mechanisms, which has substantial implications for understanding risks for poor pregnancy outcomes and evaluating the benefit of iron supplementation in pregnancy. This study is the first longitudinal study to demonstrate a temporal relationship between antenatal iron deficiency and improved birth outcomes. These findings suggest that iron supplementation needs to be integrated with other strategies to prevent or treat infections and undernutrition in pregnancy to achieve substantial improvements in birth outcomes.

**Electronic supplementary material:**

The online version of this article (10.1186/s12916-018-1146-z) contains supplementary material, which is available to authorized users.

## Background

Low birth weight (LBW; birth weight < 2500 g) and preterm birth (PTB; <37 weeks gestation) are major contributors to infant mortality and chronic childhood morbidity. LBW is associated with >80% of infant deaths [1]. Understanding factors that moderate or exacerbate the burden of LBW and PTB is, therefore, an important global health priority. Poor nutrition, anaemia and infectious diseases are common among pregnant women globally, particularly in resource-limited settings, and are important contributors to LBW. Iron deficiency (defined by the World Health Organisation [WHO] as ferritin < 15 μg/L [2]) is a common feature of undernutrition and a major cause of anaemia and it contributes to an estimated global burden of 600,000 perinatal and 100,000 maternal deaths per year [3]. Many regions with a high burden of undernutrition and anaemia are also endemic for malaria and approximately 125 million women living in malaria-endemic areas become pregnant each year [4, 5]. Malaria contributes to anaemia by causing haemolysis and dyserythropoiesis and it is also a major cause of LBW and PTB [6, 7].

Despite the high prevalence of undernutrition and anaemia among pregnant women in resource-limited and malaria-endemic settings, there is limited knowledge on the contribution of iron deficiency and other specific preventable deficiencies to LBW and poor pregnancy outcomes. To reduce the burden of anaemia and poor pregnancy outcomes, the WHO recommends that all pregnant women receive daily iron and folate supplements (30–60 mg of elemental iron plus 400 μg of folic acid), in addition to “measures to prevent, diagnose and treat malaria” in malaria-endemic areas [8]. However, there are concerns regarding the value of antenatal iron supplementation in malaria-endemic areas due to reports of harmful interactions between iron and malaria [9]. Moreover, the link between iron deficiency and poor pregnancy outcomes in malaria-endemic and tropical regions, and the value of iron supplementation on improving birth outcomes, is unclear.

Iron deficiency has been associated with reduced high-density *Plasmodium falciparum* parasitaemia, associated clinical symptoms of malaria and reduced placental *P. falciparum* infection in case–control and cross-sectional studies of pregnant women living in malaria-endemic areas [10]. Iron supplements given to children in malaria-endemic areas have been associated with increased parasitaemia, risk of clinical malaria and risk of severe illness and death (not specific to malaria) in some controlled trials [11, 12]. However, clinical trials have shown inconsistent associations between daily iron supplementation and risk of peripheral or placental *P. falciparum* infection in Africa [10, 13–15]. One study reported an increased risk of *P. vivax* after supplementation in Asia [10]. There is also no clear evidence for the association between iron supplementation and birth outcomes in pregnant women living in areas where they are at risk of malaria [10, 13–16]. A recent trial in west African pregnant and non-pregnant women found an increased rate of adverse events among those receiving iron supplementation [17]. These issues are compounded by a lack of understanding of how iron deficiency, anaemia and malaria may interact to influence birth outcomes. This knowledge is essential to the evidence base of iron supplementation programmes in malaria-endemic and resource-limited settings where there is a high burden of infectious diseases and poor nutrition.

The objectives of this study were to determine the association between iron deficiency and birth outcomes, and to quantify how malaria mediated these associations, in a longitudinal study of pregnant women in a malaria-endemic area of Papua New Guinea (PNG), which has the largest population at risk of anaemia, undernutrition malaria and poor birth outcomes in the South-West Pacific region.

## Methods

### Study design

Pregnant women (*n* = 470) were enrolled into a longitudinal study of malaria in pregnancy in Madang Province, PNG, conducted through the PNG Institute of Medical Research (Additional file 1: Methods) [18]. Briefly, study participation occurred in parallel with clinic attendance at the first antenatal visit, at 30–34 weeks’ gestation and at delivery. Pregnant women were enrolled at their first antenatal consultation at Alexishafen Health Centre between September 2005 and October 2007. At enrolment and delivery, 5 ml of peripheral blood was collected, and stored at -20 °C or -70 °C. Gestational age at enrolment was estimated from fundal height (cm) and haemoglobin was measured using a HemoCue haemoglobinometer (Hemocue, Ängelholm, Sweden). Women with haemoglobin concentrations < 5 g/dL were referred for appropriate care and were not enrolled in the study. All women were prescribed ferrous sulfate 270 mg and folic acid 0.3 mg daily, according to local policy; women with Hb 5–7 g/dL were prescribed two tablets daily. Intermittent preventative treatment in pregnancy with sulfadoxine-pyrimethamine (IPTp-SP; 1500 mg/75 mg respectively) was recommended to all women according to national guidelines. At delivery, intervillous placental blood and placental tissue samples were collected. Birth weight was measured using SECA baby scales and gestational age was estimated from Ballard scores performed by the study nurse, following training by a paediatrician [18]. Ethical approval was granted by the PNG Medical Research Advisory Council and the Alfred Health Human Research Ethics Committee, and written informed consent was obtained from all study participants. Of the 470 women enrolled into the initial study [18], 376 women completed follow-up to delivery and 97 women were excluded from the current analysis (5 multiple births; 43 newborns were not seen within 3 days after delivery; 1 data entry error; 31 with insufficient blood sample; and 17 women with missing birth outcome data) (Additional file 1). Therefore, 279 women were included in this analysis (Table 1). Included and excluded women did not differ in the distribution of enrolment variables (Additional file 1: Table S1).

**Table 1:** Participant characteristics, malaria parameters, iron markers and delivery outcomes

Variable	Mean [SD], range; or median {IQR}, range; or *N* (%)
Participant characteristics at enrolment^1^	
Maternal age, years (*N* = 268)	24 [17], 16–49
Mid upper arm circumference, cm (*N* = 270)	22.4 [1.8], 12–30
Primigravidae	106/279 (38)
Estimated gestational age, weeks (*N* = 276)	25.3 [4.2], 7–36
Education	
None or primary	154/275 (56)
Secondary+	121/275 (44)
Current smoker (yes)	56/278 (20)
Used bed net last night (yes)	200/264 (76)
Clinical history of fever, chills or headache in past 7 days (yes)	67/276 (24)
Palpable spleen (yes)	51/262 (19)
Malariometrics and iron status at enrolment	
*Plasmodium* spp. infection detectable by PCR	185/279 (66)
*Plasmodium* spp. infection^2^	98/279 (35)
*P. falciparum*	93/279 (33)
*P. vivax*	9/279 (3)
Haemoglobin, g/dL (*N* = 279)	8.5 [1.4], 5.3–12.8
Ferritin, μg/L (*N* = 279)	8.2 {4.6–17.5}, 2.4–121.3
Iron deficient (ferritin < 15 μg/L)	199/279 (71)
CRP, mg/L (*N* = 258)^3^	5.15 {1.80–11.72}, 0.18–255.65
High CRP (> 10 mg/L)	90/279 (32)
Malariometric and birth outcomes at delivery	
Placental *Plasmodium* spp. infection^2^	39/223 (17)
*P. falciparum*	34/223 (15)
*P. vivax*	6/223 (3)
*Plasmodium* spp. infection^2^	37/274 (13)
*P. falciparum*	31/274 (11)
*P. vivax*	6/274 (2)
Haemoglobin, g/dL (*N* = 270)	9.2 [1.7], 4.7–14.2
Birth weight, grams (*N* = 279)	2857 [452], 1400–4500
Low birth weight (< 2500 g)	47/279 (17)
Gestational age at delivery, weeks (*N* = 279)	38 {37–40}, 28–42
Preterm birth (<37 weeks gestation)	62/279 (22)

^1^*N* = 279

^2^*Plasmodium* spp. infection detectable by light microscopy of Giemsa-stained thick and thin blood films, unless otherwise specified. Four women had a mixed infection at enrolment (*P. falciparum* and *P. vivax*), one woman had mixed placental infection and one woman had mixed peripheral infection at delivery

^3^21 measurements were outside the assay standard curve so were excluded from the CRP concentration descriptive analysis but classified as >10 mg/L for high CRP descriptive analysis

*CRP* C-reactive protein, *IQR* interquartile range, *PCR* polymerase chain reaction, *SD* standard deviation

#### Exposures and outcomes

Exposures of interest were iron stores (ferritin) and iron deficiency (ferritin < 15 μg/L) at enrolment. Birth outcomes of interest were birth weight (grams), LBW (<2500 g) and PTB (<37 weeks gestation). Malaria outcomes of interest were peripheral *P. falciparum or P. vivax* spp. infection [by polymerase chain reaction (PCR) or light microscopy] and placental *P. falciparum* infection (detected by light microscopy or placental histology).

#### Laboratory procedures

Peripheral and placental *Plasmodium* spp. infection was determined by light microscopy of Giemsa-stained thick and thin blood films, and PCR, as previously described [18]. Among women who completed follow-up to delivery, ferritin concentrations were measured in serum samples (collected at enrolment) by established ELISA with reference controls (Immunology Consultants Laboratory Immunoperoxidase Assay). C-reactive protein (CRP) levels were determined using established ELISA kits with reference controls (Elisakit). The ferritin and CRP intra-assay and inter-assay coefficients of variation were all <10%.

### Statistical analysis

Changes in *Plasmodium* spp. prevalence and haemoglobin within an individual woman between enrolment and delivery were quantified using McNemar’s chi-square test or Student’s paired *t*-test, respectively. Associations between ferritin (transformed to log base 2) and iron deficiency and birth outcomes (birth weight, LBW and PTB) were estimated using multivariable linear and logistic regression, respectively. Confounding variables identified a priori [gravidity, maternal education, maternal mid upper arm circumference (MUAC) at enrolment (as a marker of undernutrition), maternal smoking and gestational age] were adjusted for in multivariable regression models. Red blood cell genetic polymorphisms (SAO, CR1 and α-thalassaemia) were not included in the models, as we have previously shown in this cohort that they are not associated with risk of *Plasmodium* infection or birth weight [18]. Newborn sex, a predictor of birth outcomes, was also included in the multivariable linear regression models to potentially reduce the residual standard deviation, and thus increase the precision of our parameter estimates for iron exposures. Effect modification by gravidity (primigravid/multigravid) was investigated by including an interaction term between iron exposures and gravidity; *p* values for interactions were derived from likelihood ratio tests comparing models including and excluding interaction terms. We performed sequential mediation analyses to estimate marginal natural direct and indirect effects for the association between iron deficiency and LBW mediated through: (1) peripheral infection detected by light microscopy or PCR, (2) placental *P. falciparum* infection detected by light microscopy or histology, and (3) anaemia, by calculating potential outcomes from logistic models with inverse probability weighting to achieve balance in the iron deficiency groups in terms of the confounders (gravidity, maternal education, maternal mid-upper arm circumference at enrolment, gestational age). We obtained 95% confidence intervals (CIs) for marginal natural direct effects and marginal natural indirect effects by bootstrapping using the percentile method and 1000 replications. All statistical analyses were performed using Stata Version 13 (StataCorp, College Station, TX, USA). 

## Results

### Iron deficiency and malaria in the study population

At enrolment (first antenatal visit), the median maternal age was 24 years (interquartile range, IQR 21–28), the mean estimated gestational age was 25 weeks (standard deviation, SD 4.2) and 62% of women were multigravidae. Reported use of a bed net (to prevent malaria) during the night before enrolment was 76%. For clinical symptoms, 24% of women had a history of fever, headache or chills within the 7 days prior to enrolment, and 19% had a palpable spleen (Table 1). *Plasmodium* spp. infection at enrolment was common; 35% and 66% of women had peripheral *Plasmodium* spp. parasitaemia detectable by light microscopy and PCR, respectively. The majority (95%) of infections were *P. falciparum.*

At enrolment, haemoglobin levels were generally very low (mean 8.5 g/dL, SD 1.4) and the prevalence of anaemia was very high. Overall, 95% (266/279) of women had anaemia (Hb < 11 g/dL), 61% (171/279) had moderate anaemia (Hb < 9 g/dL) and 12% (33/279) had severe anaemia (Hb < 7 g/dL). Furthermore, ferritin concentrations were low (median 8.2 μg/L; IQR 4.6–17.5) and iron deficiency (defined as ferritin < 15 μg/L according to the WHO) was present in 71% of women at enrolment (Table 1). The prevalence was 62% and 77% in primigravid and multigravid women respectively. Elevated CRP can be a marker of an acute phase response that may also raise ferritin levels and potentially lead to misclassification of iron-deficient women as iron-replete. In our study, elevated CRP (>10 mg/L) was found in 46 (57%) iron-replete women and 44 (22%) iron-deficient women (*p* < 0.001). When potentially misclassified women were excluded (women with raised CRP and ferritin > 15), the prevalence of iron deficiency was 85%. Ferritin and CRP levels were only weakly correlated (Spearman’s rho, ρ = 0.245; 95% CI 0.127, 0.357; *p* < 0.001). There was no correlation between haemoglobin at enrolment and ferritin at enrolment (ρ = 0.035; 95% CI –0.083, 0.152; *p* = 0.562), and no association between iron deficiency and anaemia at enrolment, suggesting that multiple factors contributed to anaemia, aside from iron deficiency. We found that 96% (77/80) of iron-replete and 95% (189/199) of iron-deficient women were anaemic (*p* = 0.648), 69% (55/80) of iron-replete and 58% (116/199) of iron-deficient women had moderate anaemia (*p* = 0.105), and 9% (7/80) of iron-replete and 13% (26/199) of iron-deficient women had severe anaemia (*p* = 0.313).

At delivery, 13% of women had peripheral parasitaemia detectable by light microscopy (a significant decrease from enrolment, *p* < 0.001), and mean haemoglobin was 9.2 g/dL (SD 1.7), a significant increase from enrolment, with a mean difference of −0.66 (95% CI –0.86, − 0.45; *p* < 0.001) (Table 1)*.* Placental parasitaemia was present in 17% by light microscopy (18% and 17% in primigravidae and multigravidae, respectively) (Table 1). 

### Associations between iron deficiency and birth outcomes

Mean birth weight in the cohort was 2857 g (SD 452 g) and median (IQR) gestational age was 38 weeks (37–40 weeks). Poor pregnancy outcomes were common, with 17% being LBW and 22% of babies born preterm (<37 weeks estimated gestation) (Table 1). Surprisingly, lower ferritin levels and iron deficiency were associated with higher mean birth weights in multivariable regression analyses (Table 2). For every twofold increase in ferritin, the estimated adjusted mean decrease in birth weight was −63 g (95% CI –103, −123; *p* = 0.002), and iron-deficient women gave birth to newborns that were 230 g heavier than newborns of iron-replete women (95% CI 118, 342; *p* < 0.001; Table 2). There was evidence of effect modification by gravidity, whereby the reduction in mean birth weight associated with higher iron stores was greatest in primigravidae (Table 2). Among primigravidae, birth weight was 351 g higher among iron-deficient (95% CI 188, 514), compared to iron-replete women (*p* < 0.001), whereas among multigravidae, birth weight was 125 g higher among iron-deficient women (95% CI –28, 277; *p* = 0.108; Table 2).

**Table 2:** Associations between iron deficiency and birth outcomes and effect modification by gravidity

Birth weight (grams)			
Iron stores	Unadjusted mean difference (95% CI); *p*	Adjusted mean difference (95% CI); *p*	
Ferritin, μg/L (log_2_)^1^	−72 (−113, −30); 0.001	−63 (−103, −23); 0.002	
		Primigravid	Multigravid
		−114 (−173, −55); <0.001	−20 (−74, 33); 0.457
Iron deficiency			
Iron replete	Reference group	Reference group	
Iron deficient	204 (89, 320); 0.001	230 (118, 342); <0.001	
		Primigravid	Multigravid
		351 (188, 514); <0.001	125 (−28, 277); 0.108
Low birth weight (<2500 g)			
Iron stores	Unadjusted OR (95% CI); *p*	Adjusted OR (95% CI); *p*	
Iron deficiency			
Iron replete	Reference group	Reference group	
Iron deficient	0.34 (0.18, 0.65); 0.001	0.32 (0.16, 0.64); 0.001	
		Primigravid	Multigravid
		0.26 (0.10, 0.66); 0.005	0.42 (0.15, 1.20); 0.105
Preterm birth (<37 weeks)			
Iron stores	Unadjusted OR (95% CI); *p*	Adjusted OR (95% CI); *p*	
Ferritin, μg/L (log_2_)^1^	1.13 (0.91, 1.41); 0.254	1.06 (0.84, 1.35); 0.605	
		Primigravid	Multigravid
		1.20 (0.86, 1.66); 0.280	0.93 (0.65, 1.33); 0.681
Iron deficiency			
Iron replete	Reference group	Reference group	
Iron deficient	0.60 (0.33, 1.10); 0.098	0.57 (0.30, 1.09); 0.089	
		Primigravid	Multigravid
		0.39 (0.16, 0.97); 0.042	0.86 (0.32, 2.30); 0.767

Multivariable models including confounding variables (gravidity, gestational age, education, mid-upper arm circumference and smoking), and sex of the newborn (linear models only). ^1^Ferritin transformed to log base 2 due to positively skewed distribution; coefficients are, therefore, for the absolute or relative change in outcome associated with each twofold increase in ferritin concentration. *p* values for gravidity interaction parameters are 0.019 (ferritin, birth weight), 0.042 (iron deficiency, birth weight), 0.500 (iron deficiency, LBW), 0.299 (ferritin, PTB) and 0.236 (iron deficiency, PTB)

*CI* confidence interval, *LBW* low birth weight, *OR* odds ratio, *PTB* preterm birth

Iron deficiency was associated with a 68% reduction in the odds of LBW (adjusted odds ratio, aOR = 0.32; 95% CI 0.16, 0.64; *p* = 0.001), and a 43% reduction in the odds of preterm birth, although the association with PTB had weak statistical significance (aOR = 0.57; 95% CI 0.30, 1.09; *p* = 0.089). An analysis of effect modification also showed that the magnitudes of effect between iron deficiency and LBW were highly significant and greater in primigravidae (LBW aOR 0.26; 95% CI 0.10, 0.66; *p* = 0.005) than in multigravidae (LBW aOR 0.42; 95% CI 0.15, 1.20; *p* = 0.105; Table 2). An analysis of effect modification also showed that the association between iron deficiency and PTB was restricted to primigravidae (aOR 0.39; 95% CI 0.16, 0.97; *p* = 0.042) compared to multigravidae (aOR 0.86; 95% CI 0.32, 2.30; *p* = 0.767; Table 2). Additionally, we performed analyses excluding iron-replete women who had evidence of inflammation (ferritin >15 μg/ml and CRP > 10 mg/L), that is women who may have been misclassified as iron replete due to elevated ferritin resulting from an acute phase response. This did not alter our results and a similar association between iron deficiency and birth weight was found (Additional file 1).

#### Investigating iron deficiency, malaria and anaemia as causes of LBW using mediation analyses

To investigate the causes of the association between iron deficiency and LBW further, we explored the association between iron deficiency and anaemia (Hb < 9 g/dL), peripheral malaria infection and placental malaria and whether these variables mediated the association between iron deficiency and LBW. Iron deficiency was associated with a 48% reduction in moderate anaemia at enrolment (aOR 0.52; 95% CI 0.27, 0.97; *p* = 0.040, adjusted for gravidity, education, smoking, MUAC and gestational age), reflective of multiple contributors to anaemia in the study population. Iron deficiency was also associated with a 56% reduction in peripheral blood parasitaemia at enrolment as detected by light microscopy (aOR = 0.44; 95% CI 0.25, 0.79; *p* = 0.006) and a 67% reduction in PCR-detected infection (aOR 0.33; 95% CI 0.17, 0.67; *p* = 0.002), but no association was found with placental malaria (aOR = 0.85; 95% CI 0.38, 1.90; *p* = 0.695). However, these associations were confounded by women potentially misclassified as iron replete. After excluding these potentially misclassified women, there were no significant protective associations between iron deficiency and anaemia (aOR = 0.77; 95% CI 0.33, 1.79; *p* = 0.543), peripheral parasitaemia (light microscopy aOR 1.47; 95% CI 0.59, 3.68; *p* = 0.406; PCR aOR 0.66; 95% CI 0.28, 1.56; *p* = 0.343) or placental malaria (aOR = 1.60; 95% CI 0.43, 5.87; *p* = 0.480).

**Fig. 1:**
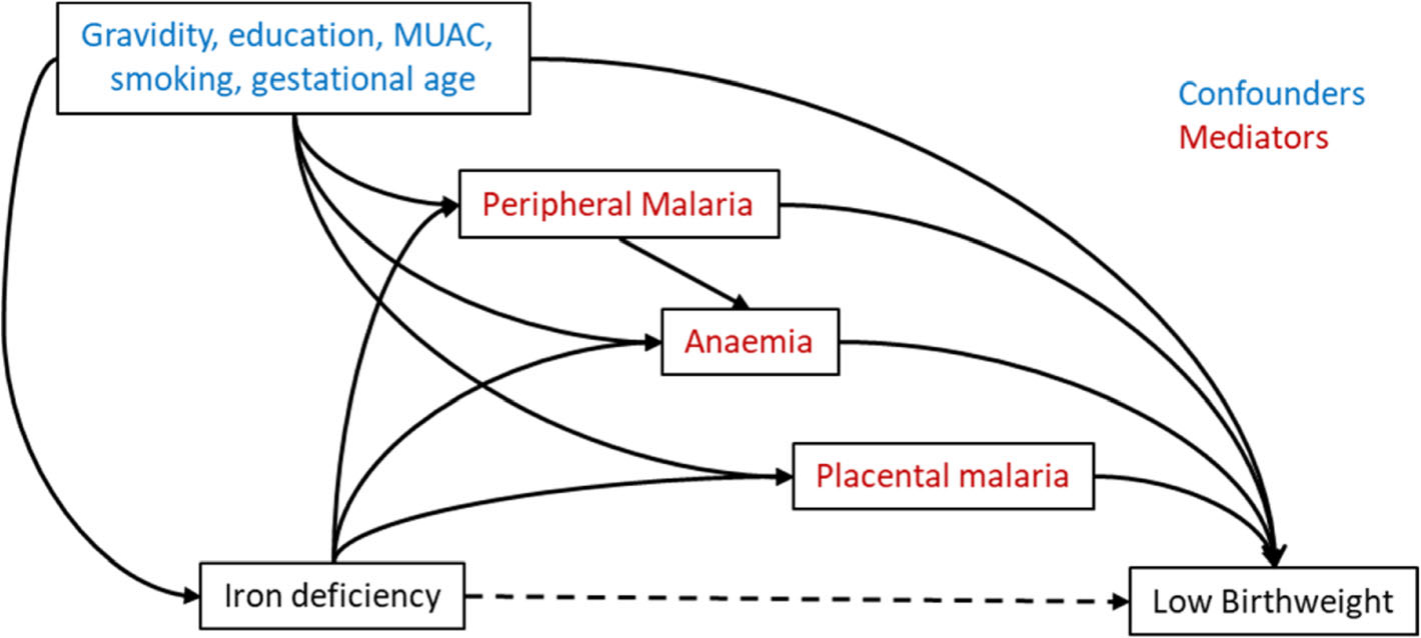
Directed acyclic graph for the mediated association between iron deficiency and low birth weight. Sequential mediation analyses were performed to assess the effects of iron deficiency on low birth weight, directly or mediated through malaria at enrolment by light microscopy, moderate anaemia at enrolment (Hb < 9 g/dL) and placental malaria at delivery by light microscopy. Gravidity, maternal education, mid-upper arm circumference, gestational age and smoking were considered confounders. The results of the analyses are shown in Table 3. MUAC mid upper arm circumference

We used sequential mediation analyses to assess the effects of iron deficiency on LBW, either directly or mediated through placental *P. falciparum* malaria, anaemia or peripheral malaria at enrolment. Sequential mediation analyses were performed to quantify the proportion of the observed protective effect of iron deficiency on LBW that was mediated through malaria and anaemia, accounting for potential confounders (maternal education, MUAC, gravidity, smoking and gestational age) (Table 3). The mediators were malaria at enrolment by light microscopy, moderate to severe anaemia at enrolment (Hb < 9 g/dL), placental malaria at delivery by light microscopy (directed acyclic graph shown in Fig. 1). The mediation analyses are sequential because there were multiple mediators that are not independent of each other (Fig. 1). In sequential mediation analyses, mediation through placental malaria only can be determined as it immediately precedes the outcome (LBW). Malaria detected at enrolment precedes other mediators of interest (anaemia and placental malaria) and therefore, mediation solely through malaria detected at enrolment cannot be determined, only what is mediated through malaria at enrolment *and* anaemia *and* placental malaria.

**Table 3:** Mediation of association between iron deficiency and low birth weight

Mediating variables	Natural direct effect, risk ratio (95% CI)	Natural indirect effect, risk ratio (95% CI)	Proportion indirect
All women			
Placental malaria only	0.47 (0.25, 0.83)	0.92 (0.63, 1.25)	7%
Anaemia and placental malaria	0.45 (0.25, 0.79)	0.88 (0.60, 1.18)	10%
Malaria, anaemia and placental malaria	0.44 (0.25, 0.79)	0.87 (0.69, 1.39)	12%
Sensitivity analyses*			
Placental malaria only	0.34 (0.17, 0.79)	1.09 (0.67, 1.80)	4%
Anaemia and placental malaria	0.33 (0.17, 0.78)	1.07 (0.63, 1. 86)	4%
Malaria, anaemia and placental malaria	0.33 (0.17, 0.78)	1.06 (0. 65, 1.84)	3%

Numbers are risk ratios (95% confidence interval). Natural direct effect is the effect of iron deficiency on birth outcome, not mediated through the specified mediators. Natural indirect effect is the effect of iron deficiency on birth outcome, mediated through the specified mediator.

*CI* confidence interval

*Women potentially misclassified as iron deficient (ferritin > 15 and C-reactive protein > 10) are excluded from analysis (*N* = 46)

Interestingly, there was evidence for a substantial direct protective effect of iron deficiency on risk of LBW that was not mediated through protection against placental malaria, anaemia or malaria present in peripheral blood (risk ratio = 0.44; 95% CI 0.25, 0.79). In contrast, only 7% of the association between iron deficiency and LBW was mediated through placental *P. falciparum* malaria and only 12% was mediated through pathways that included peripheral malaria infection, which included indirect pathways via anaemia and placental malaria (Table 3). Similar effects were seen in sensitivity analyses whereby women with raised CRP levels who may have been misclassified as iron replete (ferritin >15 μg/ml and CRP > 10 mg/L) were excluded from analyses (Table 3). Furthermore, similar associations were found in analyses investigating the mediation through malaria and anaemia detected at the second antenatal visit (30–34 weeks gestation) and/or placental malaria defined by placental histology (Additional file 1). In summary, these analyses suggest that the protective association of iron deficiency on LBW is largely mediated through mechanisms independent of malaria and anaemia.

## Discussion

LBW and PTB are major risk factors for infant morbidity and mortality and identifying factors that contribute to these risks, or protect against them, is crucial for advancing child health globally. Furthermore, concerns regarding potentially harmful malaria–iron interactions have raised questions about the appropriateness of antenatal iron supplementation for anaemia in malaria-endemic populations [9]. Here, we provide the first evidence of a protective association of maternal iron deficiency on LBW, and to a lesser extent PTB, in a malaria-endemic area where iron deficiency and anaemia are common, and elucidated the potential effect of malaria–iron interactions on birth outcomes. Iron deficiency was associated with substantially reduced risks of LBW, and this beneficial effect appeared largely mediated through mechanisms that are independent of an interaction between iron deficiency and malaria. The apparent protective effect of iron deficiency was greatest in primigravid women, a high-risk group who are more susceptible to, and more severely affected by, a range of infectious diseases [19].

We demonstrated that the associations between iron deficiency and malaria infection were confounded by the presence of an acute phase response (raised CRP). Iron deficiency was associated with reduced malaria infection when the analysis included all women. However, when women who were potentially misclassified as iron replete (ferritin> 15 but raised CRP levels) were excluded, no protective association was found. Women with malaria are more likely to have raised CRP and therefore, more likely to be misclassified as iron replete. As a result, the analyses would more likely demonstrate an apparent protective effect of iron deficiency unless these effects are considered in analyses. Previously published analyses of cross-sectional and case–control studies of associations between iron deficiency and peripheral and placental *P. falciparum* infection of women [10] have included these potentially misclassified women, which may have biased results towards finding an association between iron deficiency and reduced malaria risk, or increased the magnitude of the protective effect. There is some biological plausibility to protective associations of iron deficiency, as in vitro studies have shown that iron deficiency can limit the development of blood-stage infection by starving the parasite of exoerythrocytic forms of iron [20–23], impair merozoite invasion and propagation [24], or increase the immune-mediated clearance of infected erythrocytes [25, 26]. However, a direct causal mechanism between iron deficiency and placental malaria independent of the aforementioned reductions in peripheral infection is yet to be proposed and we found no association between iron deficiency and placental malaria in this study.

A strength of our study is that we used sequential mediation analysis as a valuable approach to estimate the contribution of potential causal relationships. This is the first analysis to quantify the mediation of the effect of iron deficiency on birth outcomes through malaria (both peripheral and placental) and anaemia. This is also the first mediation analysis of the effect of iron deficiency on birth outcomes to account for both exposure-outcome and mediator-outcome confounders, which is a source of bias in traditional mediation analysis [27]. Importantly, we found that only 12% of the protective effect of iron deficiency on LBW was mediated through malaria or anaemia. Iron deficiency was not significantly associated with anaemia or peripheral or placental malaria, which are key determinants of LBW and PTB in this population [18]. This may explain the lack of mediation through placental malaria in this cohort study and further studies using sequential mediation analyses are warranted in other populations. After accounting for mediation through peripheral *Plasmodium* spp. infection and placental *P. falciparum* infection, we found that iron deficiency was still associated directly with a >50% reduction in the odds of LBW. Host iron is essential to a range of other bacteria, parasites and viruses, and iron deficiency may also inhibit the development of these pathogens [28–30]. Therefore, reductions in infectious pathogens due to iron deficiency may explain the association between iron deficiency and improved birth outcomes in our study. In PNG, there is a high burden of disease from bacterial and viral pathogens, including respiratory, gastrointestinal and sexually transmitted infections [31], that may be influenced by iron status [30]. In PNG, a higher prevalence of these infections is also found in primigravid compared to multigravid women [32], which may also explain why larger magnitudes of the effect of iron deficiency on birth outcomes were observed in high risk primigravid women. Other infections may also be important. A recent study of iron supplementation in non-pregnant African women found higher rates of treatment for gastrointestinal infections in women receiving iron [17]. Further studies are needed to understand the mechanisms by which iron deficiency leads to improved birth weight, and this knowledge would help identify appropriate strategies to reduce LBW while also reducing the burden of iron deficiency and anaemia. Examining the effects of iron deficiency on other infectious pathogens common in pregnancy, together with their interactions with malaria and anaemia, is a priority for future studies.

This study is the first longitudinal study globally to demonstrate a temporal relationship between antenatal iron deficiency and birth outcomes, and together with the sequential mediation analyses, provides significant causal evidence for the associations observed. In PNG, malaria transmission is high and both *P. falciparum* and *P. vivax* are endemic. The majority of infections (>95%) in our study were *P. falciparum* and therefore, our findings may be more broadly applicable to other high *P. falciparum* transmission areas, such as sub-Saharan Africa. However further studies are required to determine the consistency of our findings and the effect size across areas of varying prevalence of iron deficiency, malaria and other infections and in areas where the relative contribution of the multiple factors that cause iron deficiency differs. Research that identifies populations or settings where iron deficiency associations with birth weight are strongest would be valuable in guiding public health policy. In our study, we experienced a ~40% loss to follow-up; however, given that the characteristics of the women lost to follow-up did not differ from those of the included women (Additional file 1), the loss is unlikely to have affected the internal validity of the study. While the specific causes of iron deficiency have not been defined, these are likely to include low dietary intake as well as infections such as hookworm and malaria that can impact iron absorption or utilisation.

Iron deficiency was defined according to the WHO standard definition (ferritin < 15 μg/L for non-pregnant women) [2]. Ferritin is also an acute phase protein that may increase during inflammation independently of iron stores [33]. Previous cross-sectional and case–control studies of iron deficiency and malaria in pregnancy either did not measure inflammation or reported that a proportion of women had inflammation and their reported associations may have been confounded [34, 35]. In our study, we performed sensitivity analyses to show that the protective associations observed between iron deficiency and birth weight were not confounded by the presence of inflammation. In the absence of bone marrow biopsies, ferritin remains the gold standard to assess iron deficiency [36], and although other markers of iron deficiency have been explored, such as the soluble transferrin receptor, they currently lack established definitions for iron deficiency in pregnancy. Any potential bias associated with misclassification of true iron status with the use of ferritin for defining iron deficiency is unlikely to change the conclusions of this study given the magnitude and statistical strength of the observed associations and the biological plausibility.

## Conclusions

Our findings show that iron deficiency in pregnancy is associated with a reduced risk of LBW and PTB that is not simply explained by a potential protective effect against malaria. The mechanism(s) underlying this paradox in malaria-endemic and resource-limited settings with high burdens of infectious diseases and poor nutrition are not yet well understood but are essential to elucidate given the importance and scale of this global public health issue and that LBW and PTB are important determinants of poor infant outcomes. Our results highlight that it is essential to provide both supplementation for anaemia and effective malaria prophylaxis during pregnancy, and future implementation trials to improve the uptake of these interventions as well as trials of integrated interventions for nutrition, malaria and other infections linked with LBW and PTB are warranted. Integration of screening and prophylaxis for other infectious diseases and poor nutrition, alongside current interventions in antenatal care, may be needed to achieve substantial improvements in birth and infant outcomes.

## Additional file

Additional file 1: Supplementary methods and analyses. (DOCX 142 kb)

## Abbreviations

aOR: Adjusted odds ratio
CI: Confidence intervals
CRP: C-reactive protein
Hb: Haemoglobin
IQR: Inter-quartile range
LBW: Low birthweight
MUAC: Mid upper arm circumference
OR: Odds Ratio
PCR: Polymerase chain reaction
PNG: Papua New Guinea
PTB: Pre-term birth
SD: Standard deviation
WHO: World Health Organization
