# Supplementary material for: Iron deficiency during pregnancy is associated with a reduced risk of adverse birth outcomes in a malaria-endemic area in a longitudinal cohort study
Source: BMC Med. 2018 Sep 20;16:156. doi: 10.1186/s12916-018-1146-z (PMC6149228; doi:10.1186/s12916-018-1146-z)
Supplement: Supplementary file 1 — Supplementary methods and analyses. (DOCX 142 kb) [file 12916_2018_1146_MOESM1_ESM.docx]

**Additional file**

**Iron deficiency during pregnancy is associated with a reduced risk of adverse birth outcomes in a malaria endemic area in a longitudinal cohort study**

Freya J.I. Fowkes, Kerryn A. Moore, D. Herbert Opi, Julie A. Simpson, Freya Langham, Danielle I. Stanisic, Alice Ura, Christopher L. King, Peter. M. Siba, Ivo Mueller, Stephen. J. Rogerson, James G. Beeson

**METHODS:**

**Study design**

Pregnant women (n=470; age 16 years or older) were enrolled into a longitudinal study of malaria in pregnancy in Madang Province, PNG, conducted through the Papua New Guinea Institute of Medical Research ^12^, and the study was based at the Alexishafen Health Centre. The health centre is located on the north coast of PNG and provides care predominantly for a rural population. The area experiences year-round malaria transmission; *P. falciparum* and *P. vivax* are the predominant causes of malaria, but *P. ovlae* and *P. malariae* also occur. Study participation occurred in parallel with clinic attendance at first antenatal visit, 30–34 weeks’ gestation and delivery. Pregnant women were enrolled at their first antenatal consultation at Alexishafen Health Centre between September 2005 and October 2007. Upon enrolment, basic clinical, malaria exposure (bed net use and residence), and demographic data were collected onto case report forms. Participants were asked about history of fever, headache and chills within the previous seven days, or other illnesses. At enrolment and delivery, 5ml of peripheral blood was collected, separated into serum, plasma and cell pellets, and stored at -20^o^C or -70^o^C. Gestational age at enrolment was estimated from fundal height (cm), and temperature was measured. Haemoglobin was measured using a HemoCue haemoglobinometer (Hemacue, Ängelholm, Sweden). Women with haemoglobin concentrations <5 g/dL were referred for appropriate care and were not enrolled in the study. Ferrous sulphate 270 mg and folic acid 0.3 mg daily was recommended for all women, according to local policy. Women who had a haemoglobin concentration 5-7 g/dL were prescribed double that dose. No woman was transfused or referred for transfusion during the study. Intermittent preventative treatment in pregnancy with sulphadoxine-pyrimethamine (IPTp-SP; 1500mg/75mg respectively) was recommended to all women according to national guidelines. IPTp and iron uptake and compliance was not recorded. At delivery, birthweight was measured using SECA baby scales (within 3 days after delivery), and gestational age was estimated from Ballard scores ^12^. Intervillous placental blood samples were collected, and a 1 cm^2^ full thickness placental biopsy was collected and placed in neutral buffered formalin for placental histology. Women were also seen at 30-34 weeks’ gestation as part of antenatal care (where enrolment was before 30 weeks’ gestation), and women were screened for malaria.

Of the 470 women enrolled into the initial study^12^, 376 women completed follow-up to delivery and 97 women were excluded from the current analysis for the following reasons: 5 multiple births; 43 newborns were not seen within three days after delivery (the cut-off for assessing low birthweight); 1 data entry error; 31 with insufficient blood sample available to measure ferritin levels; 17 women had missing birth outcome data) (supplementary figure 1). Therefore, 279 women were included in this analysis (Table 1). Included and excluded women did not differ in the frequency and distribution of enrolment variables. There were no maternal or neonatal deaths in the study.

The presence of malaria infection was assessed by standard light microscopy of thick and thin blood films, and by PCR of DNA extracted from venous or placental blood samples, as described previously^12^. The presence of red blood cell genetic polymorphisms that are common in PNG (CR1 polymorphisms, South-Ease Asian ovalocytosis, alpha-thalassemia) and that may influence anaemia and susceptibility to malaria were evaluated by PCR as described^12^. However, these were not found to be significantly associated with the risk of *Plasmodium* infection or birth weight.

Ethical approval was granted by the PNG Medical Research Advisory Council and the Alfred Health Human Research Ethics Committee, and written informed consent was obtained from all participants.

**Table S1**

| **Variable** | **Included women**  Mean [SD], range;  or Median {IQR}, range; or N (%) | **Excluded women**^1^  Mean [SD], range;  or Median {IQR}, range; or N (%) | ***P***^2^ |
| --- | --- | --- | --- |
| **Participant characteristics at enrolment** | |  |  |
| Maternal age, years | 24 [17], 16-49 | 24 [17], 16-42 | 0.87 |
| Mid-Upper Arm Circumference, cm | 22.4 [1.8], 12-30 | 22.5 [1.9], 12-29 | 0.57 |
| Primigravidae | 106 (38) | 72 (37) | 0.95 |
| Estimated gestational age, weeks | 25.3 [4.2], 7-36 | 25.2 [4.5], 15-38 | 0.83 |
| Education |  |  |  |
| None/Primary | 154 (56) | 120 (64) | 0.09 |
| Secondary + | 121 (44) | 68 (36) |  |
| Current smoker (yes) | 56 (20) | 32 (17) | 0.36 |
| Used bed net last night (yes) | 200 (76) | 135 (72) | 0.39 |
| Clinical history of fever, chills, headache in past 7 days (yes) | 67 (24) | 55 (29) | 0.26 |
| Palpable spleen (yes) | 51 (19) | 35 (19) | 0.99 |
| **Malariametrics and iron status at enrolment** | |  |  |
| *Plasmodium* spp. infection detectable by PCR | 185 (66) | 122 (65) | 0.69 |
| *Plasmodium* spp. infection^3^ | 98 (35) | 63 (33) | 0.63 |
| *P. falciparum* | 93 (33) | 55 (29) | 0.3 |
| *P. vivax* | 9 (3) | 16 (8) | 0.02 |
| Haemoglobin, g/dL | 8.5 [1.4], 5.3-12.8 | 8.5 [1.4], 5-12 | 0.59 |
| Moderate anaemia | 108 (61) | 121 (63) | 0.65 |
| Ferritin, μg/L | 8.2 {4.6-17.5}, 2.4-121.3 | 9.4 {5.4-15.4}, 2.0-85.0 | 0.58 |
| Iron deficient (ferritin <15 μg/L) | 199 (71) | 49 (74) | 0.64 |

^1^ 97 women were excluded from iron deficiency analysis (5 multiple births; 43 newborns weighed more than three days after delivery; 1 data entry error; 31 with insufficient blood sample to determine ferritin concentrations; and 17 women with missing birth outcome data.

^2^ The associations between categorical variables were assessed using Pearson’s Chi-squared test, and between continuous variables by Student’s *t*-test, or the Mann-Whitney *U* test, as appropriate.

^3^ *Plasmodium* spp. infection detectable by light microscopy of Giemsa-stained thick and thin blood films, unless otherwise specified.

**Table S2.**

**Associations between iron deficiency and birth outcomes and effect modification by gravidity exluding women potentially misclassified as iron deficient (ferritin >15 and CRP>10, N = 46)**

| **Iron stores** | **Birth Outcome** | **Gravidity** | **Adjusted mean difference/OR**  **(95%CI); *p*** |
| --- | --- | --- | --- |
| Ferritin | Birthweight (grams) | All | -77 (-132, -23); 0.006 |
| Ferritin | Birthweight (grams) | PG | -125 (-206, -44); 0.003 |
| Ferritin | Birthweight (grams) | MG | -38 (-111, 36); 0.312 |
| Ferritin | Preterm (<37 weeks) | All | 1.00 (0.72, 1.39); 0.988 |
| Ferritin | Preterm (<37 weeks) | PG | 1.12 (0.72, 1.75); 0.610 |
| Ferritin | Preterm (<37 weeks) | MG | 0.87 (0.53, 1.44); 0.598 |
| Iron deficiency | Birthweight (grams) | All | 300 (144, 457); <0.001 |
| Iron deficiency | Birthweight (grams) | PG | 403 (180, 625); <0.001 |
| Iron deficiency | Birthweight (grams) | MG | 200 (-21, 420); 0.075 |
| Iron deficiency | LBW (<2500 grams) | All | 0.24 (0.10, 0.58); 0.002 |
| Iron deficiency | LBW (<2500 grams) | PG | 0.30 (0.09, 1.05); 0.059 |
| Iron deficiency | LBW (<2500 grams) | MG | 0.18 (0.05, 0.65); 0.009 |
| Iron deficiency | Preterm (<37 weeks) | All | 0.42 (0.18, 0.99); 0.048 |
| Iron deficiency | Preterm (<37 weeks) | PG | 0.25 (0.07, 0.81); 0.021 |
| Iron deficiency | Preterm (<37 weeks) | MG | 0.81 (0.20, 3.21); 0.765 |

Multivariable models including confounding variables (gravidity, gestational age, education, mid-upper arm circumference, and smoking), and sex of the newborn (linear models only).Ferritin transformed to log base-2 due to positively skewed distribution; coefficients are therefore for the absolute or relative change in outcome associated with each two-fold increase in ferritin concentration. PG, Primigravid; MG, Multigravid

**Table S3.**

| **Mediating variables** | **Natural Direct Effect, risk ratio (95%CI)** | **Natural Indirect Effect, risk ratio (95%CI)** | **Proportion indirect (%)** |
| --- | --- | --- | --- |
| **All women, with malaria by PCR** |  |  |  |
| Placental malaria only | 0.41 ( 0.20, 0.75) | 0.94 ( 0.61, 1.30) | 4 |
| Anaemia and Placental malaria | 0.39 ( 0.19, 0.71) | 0.90 ( 0.58, 1.27) | 7 |
| Malaria PCR, Anaemia and Placental malaria | 0.44 ( 0.25, 0.79) | 1.03 ( 0.66, 1.56) | -2 |
| **All women, placental histology** |  |  |  |
| Placental malaria only (histology) | 0.47 ( 0.25, 0.83) | 1.05 ( 0.75, 1.43) | -4 |
| Anaemia and Placental malaria (histology) | 0.45 ( 0.25, 0.79) | 1.00 ( 0.73, 1.37) | 0 |
| Malaria, Anaemia and Placental malaria (histology) | 0.44 ( 0.25, 0.79) | 0.98 ( 0.69, 1.39) | 1 |
| **All women, V2 anaemia, V2 malaria, placental malaria (LM)** |  |  |  |
| Placental malaria only (LM) | 0.46 ( 0.25, 0.87) | 0.90 ( 0.60, 1.19) | 9 |
| Anaemia (V2) and Placental malaria (LM) | 0.45 ( 0.26, 0.85) | 0.88 ( 0.59, 1.17) | 10 |
| Malaria (V2), Anaemia (V2) and Placental malaria (LM) | 0.44 ( 0.25, 0.79) | 0.86 ( 0.53, 1.16) | 13 |
| **All women, V2 anaemia, V2 malaria, placental histology** |  |  |  |
| Placental malaria only (hist) | 0.46 ( 0.25, 0.87) | 1.02 ( 0.67, 1.40) | -1 |
| Anaemia (V2) and Placental malaria (hist) | 0.45 ( 0.26, 0.85) | 1.00 ( 0.65, 1.40) | 0 |
| Malaria (V2), Anaemia (V2) and Placental malaria (hist) | 0.44 ( 0.25, 0.79) | 0.98 ( 0.60, 1.42) | 2 |

Numbers are risk ratios (95% confidence interval). Sequential mediation analyses were performed to quantify the proportion of the observed protective effect of iron deficiency on low birthweight that was mediated through malaria and anaemia, accounting for potential confounders (maternal education, mid-upper arm circumference, gravidity, smoking and gestational age). The mediators were malaria at enrolment (or visit 2, V2) by light microscopy, moderate-severe anaemia at enrolment or V2 (Hb<9 g/dL), placental malaria at delivery by light microscopy or placental histology (no infection vs. acute/chronic/past infection) (directed acyclic graph shown in Figure 1). At visit 2 the mean (standard deviation) of Hb was 8.92 (1.34) and 62/240 (25.8%) or women were positive by malaria by light microscopy. The mediation analyses are sequential because there were multiple mediators that are not independent of each other (Figure 1). In sequential mediation analyses, mediation through placental malaria only can be determined as it immediately precedes the outcome (low birthweight). Malaria detected at enrolment precedes other mediators of interest (anaemia and placental malaria), and therefore mediation solely through malaria detected at enrolment cannot be determined, only what is mediated through malaria at enrolment *and*anaemia *and*placental malaria.  Natural direct effect (*NDE*, the effect of iron deficiency on birth outcome, not mediated through the specified mediator/s); natural indirect effect (*NIE*, the effect of iron deficiency on birth outcome, mediated through the specified mediator/s).

**Figure S1. Participant flow throughout the study**


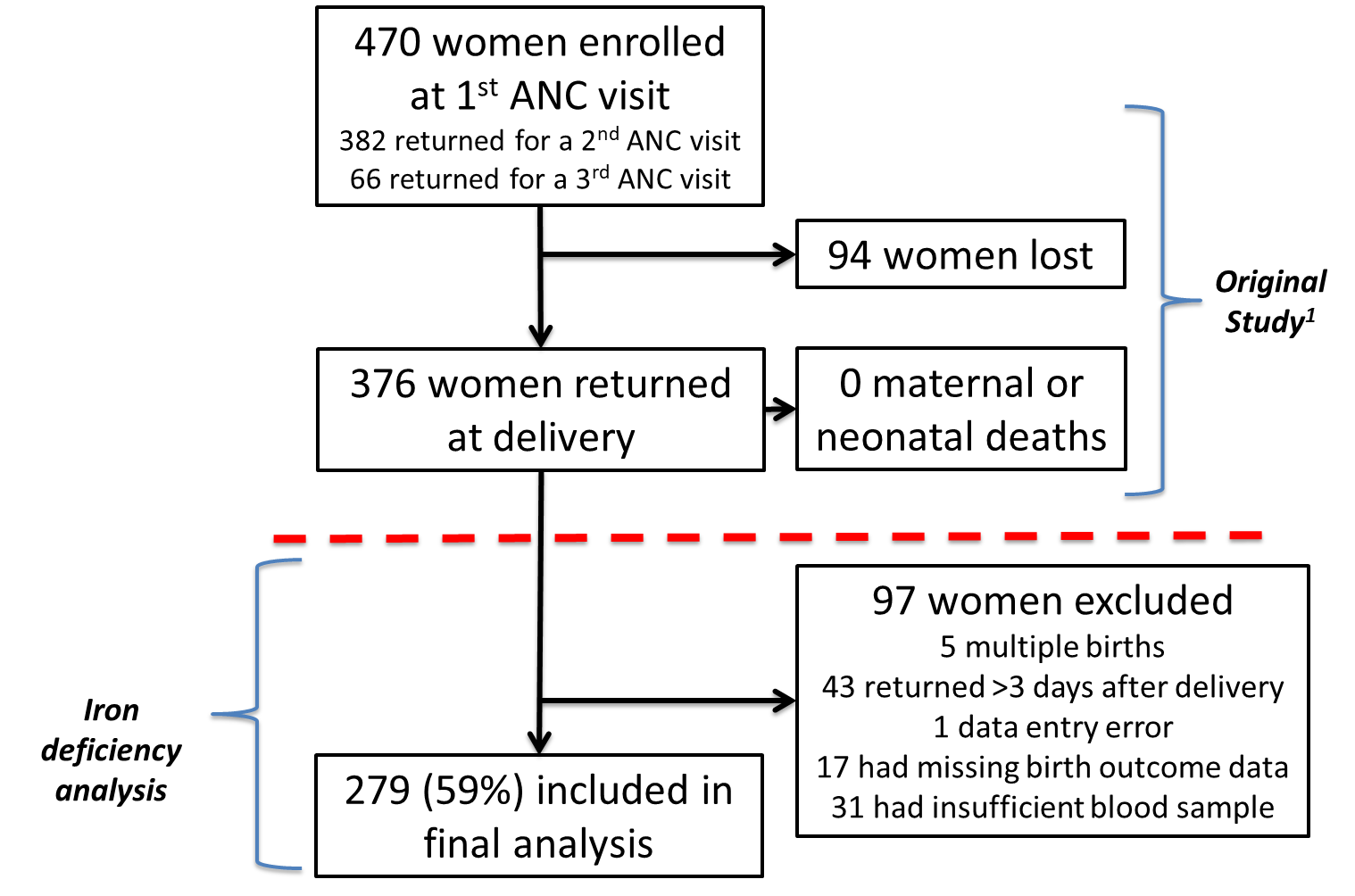


^1^ The original cohort study is published: Stanisic, D. I., K. A. Moore, F. Baiwog, A. Ura, C. Clapham, C. L. King, P. M. Siba, J. G. Beeson, I. Mueller, F. J. Fowkes and S. J. Rogerson (2015). "Risk factors for malaria and adverse birth outcomes in a prospective cohort of pregnant women resident in a high malaria transmission area of Papua New Guinea." Trans R Soc Trop Med Hyg **109**(5): 313-324. Study participation occurred in parallel with clinic attendance at first antenatal visit, 30–34 weeks’ gestation and delivery.
